# Assessing adverse effects of intra-articular botulinum toxin A in healthy Beagle dogs: A placebo-controlled, blinded, randomized trial

**DOI:** 10.1371/journal.pone.0191043

**Published:** 2018-01-10

**Authors:** Helka M. Heikkilä, Tarja S. Jokinen, Pernilla Syrjä, Jouni Junnila, Anna Hielm-Björkman, Outi Laitinen-Vapaavuori

**Affiliations:** 1 Department of Equine and Small Animal Medicine, Faculty of Veterinary Medicine, University of Helsinki, Helsinki, Finland; 2 Department of Veterinary Biosciences, Veterinary Pathology, Faculty of Veterinary Medicine, University of Helsinki, Helsinki, Finland; 3 Oy 4Pharma Ltd., Helsinki, Finland; Southern Illinois University School of Medicine, UNITED STATES

## Abstract

**Objective:**

To investigate the clinical, cytological, and histopathological adverse effects of intra-articularly injected botulinum toxin A in dogs and to study whether the toxin spreads from the joint after the injection.

**Methods:**

A longitudinal, placebo-controlled, randomized clinical trial was conducted with six healthy laboratory Beagle dogs. Stifle joints were randomized to receive either 30 IU of onabotulinum toxin A or placebo in a 1:1 ratio. Adverse effects and spread of the toxin were examined by evaluating dynamic and static weight-bearing of the injected limbs, by assessing painless range of motion and pain on palpation of joints, and by performing synovial fluid analysis, neurological examination, and electrophysiological recordings at different examination time-points in a 12-week period after the injections. The dogs were then euthanized and autopsy and histopathological examination of joint structures and adjacent muscles and nerves were performed.

**Results:**

Intra-articular botulinum toxin A did not cause local weakness or injection site pain. Instead, static weight-bearing and painless range of motion of stifle joints decreased in the placebo limbs. No clinically significant abnormalities associated with intra-articular botulinum toxin A were detected in the neurological examinations. Electrophysiological recordings showed low compound muscle action potentials in two dogs in the botulinum toxin A-injected limb. No significant changes were detected in the synovial fluid. Autopsy and histopathological examination of the joint and adjacent muscles and nerves did not reveal histopathological adverse effects of the toxin.

**Conclusion:**

Intra-articular botulinum toxin A does not produce significant clinical, cytological, or histopathological adverse effects in healthy dogs. Based on the electrophysiological recordings, the toxin may spread from the joint, but its clinical impact seems to be low.

## Introduction

Botulinum toxins are the strongest toxins known to man [1]. They are produced by the bacterium *Clostridium botulinum* in at least seven serotypes, with serotype A (BoNT/A) appearing to be the most potent [2,3]. Botulinum toxins inhibit acetylcholine release in the neuromuscular junction and in the cholinergic nerve terminals within the autonomic nervous system, which leads to paralysis of affected muscles and inhibition of secretion in affected glands [4]. These effects have been exploited in medicine, where intramuscular (IM) and subdermal BoNT injections have been used for treatment of various painful musculoskeletal conditions and hyperhidrosis already since the 1980s [5].

Osteoarthritis (OA) is one of the most common causes for disability, with a growing clinical importance in man worldwide [6]. The current pharmacological management of OA consists of topical and oral pain medication and intra-articular (IA) therapy [7,8]. Intra-articular therapy is directly targeted to the painful joint, which reduces the systemic effects of the treatment and can lead to greater pain relief than systemic pain medication [9]. This is especially beneficial for patients with chronic pain and comorbidities because such patients have an increased risk for intolerable adverse effects of oral pain medication [10]. Conventional IA treatment of OA consists of IA corticosteroids or hyaluronic acid. However, controversy exists regarding their efficacy, adverse effects, and recommendations for use [7–9,11–13]. Recently, new IA therapies, including IA BoNT/A, have attracted attention in the treatment of OA. The rationale behind the use of IA BoNT/A in the treatment of OA pain is its ability to inhibit the release of neurotransmitter substance P [14,15], calcitonin gene-related peptide [16], and glutamate [17,18], which are also involved in the sensation of pain in OA [19,20]. However in our recent study, IA BoNT/A did not affect the concentration of substance P inside a joint ([21].

Intra-articular BoNT/A has shown efficacy in relieving joint pain in controlled clinical trials in human patients suffering from OA and rheumatoid arthritis [22–25], in human patients with painful prosthetic knees [26] or adhesive capsulitis of the shoulder joint [27], and also in osteoarthritic dogs [28]. However, contradictory results have also been published [29]. In two studies in human patients with painful knee or shoulder joints, IA BoNT/A was found as effective as IA corticosteroids [24,27]. In a case series of rheumatoid and osteoarthritic patients, the pain relief lasted for 3–17 months [30], which might indicate an advantage over IA corticosteroids. Despite these promising findings, the safety of IA BoNT/A has not, to our knowledge, been thoroughly investigated.

Botulinum toxins have a fearful reputation for being the cause of a progressive neuroparalytic disease, botulism [2], which, still today, is a life-threatening condition. Traditionally, botulism has been a consequence of ingestion of BoNTs in food or production of the toxin in the gut or in a wound colonized by the bacterium [2], but it is also a concern when the toxin is used in medicine. The safety of IM and intradermal BoNT injections has been thoroughly assessed in human patients [31–33] with the conclusion that BoNT injections are safe and generally well tolerated. Despite this, generalized botulism-like syndrome due to systemic spread of the toxin has been reported after IM injection [34–39]. The toxin may spread systemically also in patients without any clinical signs [40] and cause subclinical changes, including long-lasting atrophy in the injected muscles [41,42] and degeneration and atrophy in the adjacent nerve [43]. Botulinum toxin injections are not routinely used in veterinary medicine, and therefore, their safety has not been assessed in dogs.

No serious clinical adverse effects, i.e. hospitalization, anaphylaxis, or death, were reported in humans [22–27] or in dogs [28,44] after IA BoNT/A. In human patients, the procedures for the monitoring of less serious adverse events have included interviews, manual muscle strength testing, muscle strength dynamometer measurement, and repeated neurological examinations [22–24,26]. In dogs, the monitoring has been performed with neurological examinations and owner interviews [28,44]. However, to the authors´ knowledge, no published studies have focused specifically on the adverse effects of IA BoNT/A injections. Potential systemic spread after an IA injection has also not been previously investigated.

The purpose of this study was to address the question of the safety of IA BoNT/A by investigating any clinical, cytological, and histopathological adverse effects of the toxin when directly injected into a canine joint. Also, our aim was to determine whether the toxin spreads from the joint after the IA injection. The hypothesis was that IA BoNT/A will not cause clinical, cytological, or histopathological adverse effects, but may spread from the joint.

## Materials and methods

### Ethical statement

The study was formally approved by the Committee of Experimental Animals (ESAVI-2010-04178/Ym-23) and the Finnish Medicines Agency. The study was conducted and the dogs were housed and taken care of in compliance with the Finnish legislation on animal experimentation and the directive 2010/63/EU of the European Union and Council at the Laboratory Animal Centre of the University of Helsinki. All efforts were taken to minimize suffering and distress of the dogs during the study.

### Animal model

Six laboratory Beagle dogs were studied. The dogs were healthy intact six-year-old females with a median weight of 12.5 kg (range 10.2–14.2 kg). They were assigned for euthanasia due to discontinuation of the use of beagles in research. The dogs were confirmed healthy by clinical, orthopedic, and neurologic examinations, by analysis of hematology and serum biochemistry, and by evaluation of radiographs taken from both stifle joints, hip joints, and the lumbar spine.

The study was carried out as a longitudinal, placebo-controlled, blinded, randomized trial. The dogs were randomized in a 1:1 ratio to receive 30 IU of onabotulinum toxin A (BoNT/A) into either the right or left stifle joint. The dosage of BoNT/A was based on earlier studies in human patients [22–24,26,30,45] and on our clinical experience from a previous treatment efficacy study [28]. The same volume of placebo (0.9% sterile saline) was injected into the contralateral joint. A research nurse performed the randomization of joints by drawing lottery tickets. The nurse prepared the injections and covered the syringes with non-transparent tape. The veterinarians performing the study were blinded to the treatment allocation.

The dogs were evaluated for the occurrence of clinical and cytological adverse effects and for the spread of the toxin at 24 and 72 h and at 1, 2, 4, 8, and 12 weeks after the IA injection. After 12 weeks, the dogs were euthanized and evaluated for histopathological adverse effects postmortem. The detailed schedule for the evaluations performed during the study is presented in Table 1.

**Table 1:** Schedule for examinations.

Examination	Timepoint							
	Baseline	24 h	72 h	1 W	2 W	4 W	8 W	12 W
Dynamic weight-bearing	X				X	X	X	X
Static weight-bearing	X			X	X	X	X	X
Painless range of motion of joint	X	X	X	X	X	X	X	X
Pain on palpation of joint	X	X	X	X	X	X	X	X
Neurological examination	X	X	X	X	X	X	X	X
Electrophysiological recordings	X				X	X	X	X
Synovial fluid analysis	X				X	X	X	X
Postmortem histopathological examination								X

Baseline, before injections of intra-articular botulinum toxin A and placebo; h, hour; W, week

### Evaluation of clinical adverse effects

The assessment of local weakness of the hind limbs included dynamic and static weight-bearing measurements. Injection site pain was evaluated by palpation of the stifle joints and measuring the painless range of motion in the stifle joints.

#### Dynamic weight-bearing

The dynamic weight-bearing was evaluated by calculating a symmetry index from the peak vertical forces (PVFs) of the hind limbs. The PVFs were obtained with a force platform (Kistler force plate, type 9286, Kistler Instrumente AG, Winterhur, Switzerland) and a computer software program (Aquire 7.3, Sharon Software Inc., Dewitt, MI, USA) at trot at a comfortable speed as described previously [28]. The symmetry index was calculated using the following equation: 100 [(PVF_BoNT_-PVF_Pla_) / 0.5(PVF_BoNT_ + PVF_Pla_)], where PVF_BoNT_ is the mean of the PVFs of the IA BoNT/A-injected limb and PVF_Pla_ is the mean of the PVFs of the placebo-injected limb. A symmetry index of 0 indicates perfect symmetry. A positive value means that the dog bears more weight on the IA BoNT/A-injected limb, and a negative value means that the dog bears more weight on the IA placebo-injected limb.

#### Static weight-bearing

The static weight-bearing of both hind limbs was measured with two factory-calibrated bathroom scales [46]. The hind limbs were symmetrically placed on the scales, while the front limbs were placed on a custom-made platform of the same height. A measurement was considered valid if the dog stood straight and remained still until a fixed value was obtained for both limbs. Five measurements were recorded for both limbs at each examination time-point, and their mean was used for analysis.

#### Painless range of motion of joint

The range of motion of the stifle joints was measured with a universal plastic goniometer as described previously [47]. To obtain the painless range of motion of the joints, the measurements were performed on awake dogs. A decrease in the range of motion indicates pain in the joint. Three measurements were obtained for both stifle joints at each examination time-point, and their mean was used for analysis.

#### Pain in palpation of joint

A veterinarian (HMH) evaluated pain on palpation of both stifle joints of each dog using a five-point scale from 0 to 4: 0 = no sign of pain; 1 = mild pain (dog turns head in recognition); 2 = moderate pain (dog pulls limb away); 3 = severe pain (dog vocalizes or becomes aggressive); and 4 = extreme pain (dog does not allow palpation), as in Heikkilä et al. (2014) [28].

### Evaluation of the spread of toxin

The spread of the toxin was evaluated by neurological examinations and electrophysiological recordings.

#### Neurological examination

The neurological examination included evaluation of mental status and gait, testing of postural reactions including evaluation of proprioception and wheel barrowing, testing of spinal reflexes including myotatic and withdrawal reflexes, and evaluation of cranial nerves.

#### Electrophysiological recordings

The dogs were sedated with IM dexmedetomidine (5 μg/kg) and butorphanol (0.01 mg/kg) for the recordings. Intravenous propofol was used to effect, when necessary. The electrophysiological recordings were performed with a Nicolet Viking Quest (Nicolet Biomedical Inc., Madison, WI, USA), and they consisted of electromyography (EMG) and assessment of motor nerve conduction velocities (MNCVs), amplitudes of compound muscle action potentials (CMAPs), and repetitive nerve stimulation (RNS).

Electromyography was performed bilaterally in the proximal and distal muscles of the pelvic and thoracic limbs and in the paraspinal muscles. A concentric needle electrode was used for the EMG, while a subdermal needle electrode served as a ground on the animal´s flank. Abnormalities detected included abnormal insertional activity and such spontaneous activity as fibrillations and positive sharp waves.

The motor nerve conduction velocities of the sciatic/peroneal nerves were measured bilaterally with supramaximal stimulus, as described previously [48]. The measurement was performed with two stimulating monopolar needle electrodes and three subdermal needle electrodes as the recording, reference, and ground electrodes. Amplitudes of the CMAPs were also recorded.

Repetitive nerve stimulation was performed at 3 Hz with supramaximal stimulus, stimulus duration of 0.2 ms and train of 10 stimuli. The peroneal nerves with distal stimulation sites were used for the RNS test. Compound muscle action potentials were recorded. Data analysis consisted of comparing the CMAP amplitudes and areas of subsequent potentials to the initial one. The rectal temperature of the dogs was measured during the electrophysiological recordings.

### Evaluation of cytological and histopathological adverse effects

The cytological and histopathological adverse effects were evaluated by synovial fluid (SF) analyses performed during the study and complete autopsy and histopathological examination of stifle joints and adjacent muscles and nerves postmortem.

#### Synovial fluid analysis

The dogs were sedated for the arthrocentesis as described above in the section on electrophysiological recordings. Synovial fluid samples were obtained from both stifle joints of each dog after aseptically preparing the joints. Synovial fluid was inspected visually for changes in color, viscosity, and turbidity. After this, the SF samples were put into EDTA tubes and total and differential cell counts including the percentages of neutrophils and mononuclear cells were analyzed within 30 min of the arthrocentesis. If an insufficient volume of SF was obtained, the sample was placed directly onto slides and only the differential cell count was analyzed. The percentage of neutrophils in the SF samples was used for analysis.

#### Histopathological examination

At 12 weeks, the dogs were sedated with intravenous medetomidine (0.02 mg/kg), butorphanol (0.1 mg/kg), and MK-467 (1 mg/kg), after which euthanasia was performed by intravenous propofol (10 mg/kg) and pentobarbital (50 mg/kg). The dogs underwent a complete autopsy, including histopathology of all major internal organs. In addition, the stifle joints and the adjacent muscles and nerves were specifically evaluated for histopathological adverse effects of IA BoNT/A.

The stifle joints were macroscopically evaluated immediately after euthanasia. The articular cartilage, synovial lining, and joint capsule were inspected for discoloration and changes in thickness and surface roughness. After this, both stifle joints from all dogs were dissected and immersed into 10% neutral buffered formalin for a minimum of 36 h. Thereafter, biopsies were obtained as sagittal sections from the cartilage of the weight-bearing areas of the medial and lateral femoral condyles as well as the medial and lateral tibial plateau. Soft tissue was sampled from the synovium. Samples were embedded in paraffin wax, sectioned routinely, and stained with hematoxylin and eosin and toluidine blue. The samples from bone were decalcified in EDTA solution before embedding.

Two blinded independent reviewers (HMH and PS) performed the histopathological evaluation of the cartilage and synovium biopsies as described in the OARSI Histopathology Initiative [49]. Briefly, cartilage structure was evaluated based on structural integrity and chondrocyte pathology, while the synovial structure was graded with respect to lining cell layers and presence of villous hyperplasia. The presence of inflammatory infiltrates in the synovium was also evaluated. The details of the scoring system are provided in Table 2.

**Table 2:** Histopathological grading of cartilage and synovial changes.

Variable	Classification	Characteristics	None	Local	Multifocal	Global
Cartilage structure	Structural integrity	Normal	0	0	0	0
		Fissures in upper zone	0	1	2	3
		Fissures to mid zone	0	2	4	6
		Fissures to deep zone	0	3	6	9
		Full thickness loss of cartilage	0	4	8	12
	Chondrocyte pathology	None	0	0	0	0
		Loss of cells or relative increased density	0	1	2	3
		Small cell clusters	0	2	4	6
		Large cell clusters	0	3	6	9
		Cell loss	0	4	8	12
Synovial structure	Cell layers	1–2 layers	0	0	0	0
		3–6 layers	0	1	2	3
		>6 layers	0	2	4	6
	Villous hyperplasia	None	0	0	0	0
		Short villi	0	1	2	3
		Finger-like	0	2	4	6
Synovial inflammatory cell infiltrates		None	0	0	0	0
		Mild to moderate	0	1	2	3
		Marked and diffuse	0	2	4	6

The scores for structural integrity and chondrocyte pathology were summed and used as the score for cartilage structure. The scores for cell layers and villous hyperplasia were summed and used as the score for synovial structure. The histopathological grading was modified from the OARSI Initiative [49].

The nerve and muscle biopsies were taken from the sciatic, tibial, and saphenous nerves, and the popliteal, vastus lateralis, and semimembranosus muscles bilaterally from each dog. The biopsies were fixed in 10% neutral buffered formalin, along with samples of internal organs, embedded in paraffin wax, sectioned routinely, and stained with hematoxylin and eosin.

The autopsy and the histopathological evaluation of the muscle and nerve biopsies were performed by a blinded veterinary pathologist (PS). One cross section and one longitudinal section were evaluated from each examined muscle. The muscle biopsies were evaluated for the presence of muscle cell size variation and angular and Ring fibers, characterized by a peripheral circumferential band within the muscle fiber on cross section. The nerve biopsies were evaluated for the presence of proliferating Schwann cells (Büngner bands), Wallerian degeneration, and inflammatory cell infiltrates.

### Statistical methods

Descriptive statistics and frequency tables were used to summarize the data by treatment and examination time-point. Symmetry index, static weight-bearing, and painless range of motion were analyzed with repeated measures analysis of covariance models (RM-ANCOVA). The examination time-point and baseline measurement were used as fixed terms and dog as a random term in the model used for the symmetry index. In the model for static weight-bearing and painless range of motion, the change from baseline was used as the response and treatment, time-point, injected limb (left or right), and interaction between treatment and examination time-point were included as fixed effects. The corresponding baseline measurement served as a covariate, and dog within injected limb was used as a random term.

The post-baseline neutrophil percentages in the SF samples were analyzed with a repeated measures analysis of variance (RM-ANOVA) model, where treatment, examination time-point, injected limb, and interaction between treatment and time-point were included as fixed effects and dog within injected limb as the random term.

The histopathological variables were analyzed with the following approaches. The variables synovium structure and synovium infiltrates were analyzed with cumulative logit-models with treatment and injected limb as fixed terms and dog as the random subject effect. The variables muscle cell size variation, angular and Ring fibers, Büngner bands, Wallerian degeneration, and dichotomized cartilage structure (0 vs > 0) were analyzed with logistic regression, where at least the treatment was included as a fixed term and injected limb if feasible. Dog was used as a random subject effect. The number of inflammatory cells in the nerve biopsies was analyzed with an ANOVA model, where treatment and injected limb were included as fixed terms and dog within injected limb as a random term.

The differences between treatments and examination time-points together with two-sided 95% confidence intervals (CIs) for the difference were estimated from the fitted RM-ANCOVA, RM-ANOVA, and ANOVA models using contrasts. In the logistic and cumulative logit-models, the differences between treatments were quantified with odds ratios and CIs. P-values <0.05 were considered significant. All statistical analyses were performed using SAS^®^ System for Windows, version 9.3 (SAS Institute, Cary, NC, USA).

## Results

### Evaluation of clinical adverse effects

#### Dynamic weight-bearing

**Fig 1:**
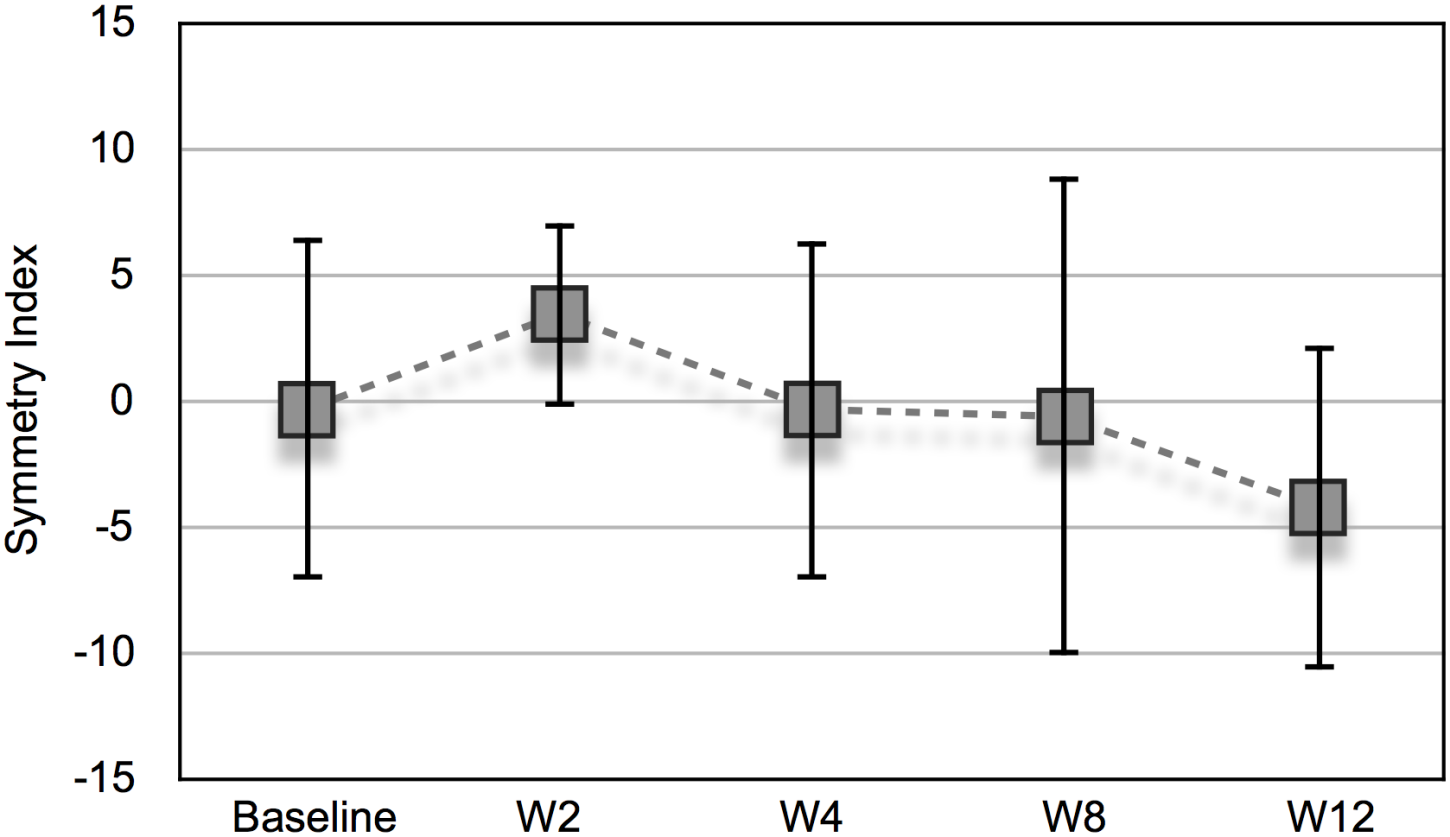
Dynamic weight-bearing after intra-articular botulinum toxin A and placebo. Symmetry indices (presented as mean and SD) of six healthy Beagle dogs were calculated from hind limb PVFs obtained with a force platform at trot. 0 = dog is moving in perfect symmetry; > 0 more weight-bearing on the IA BoNT/A injected limb; < 0 more weight-bearing on the IA placebo injected limb. Baseline, before the injections; W, week.

There was no statistically significant change from baseline in the symmetry indices in favor of either limb during the study (*P* = 0.106) (Fig 1) (S1 Table).

#### Static weight-bearing

**Fig 2:**
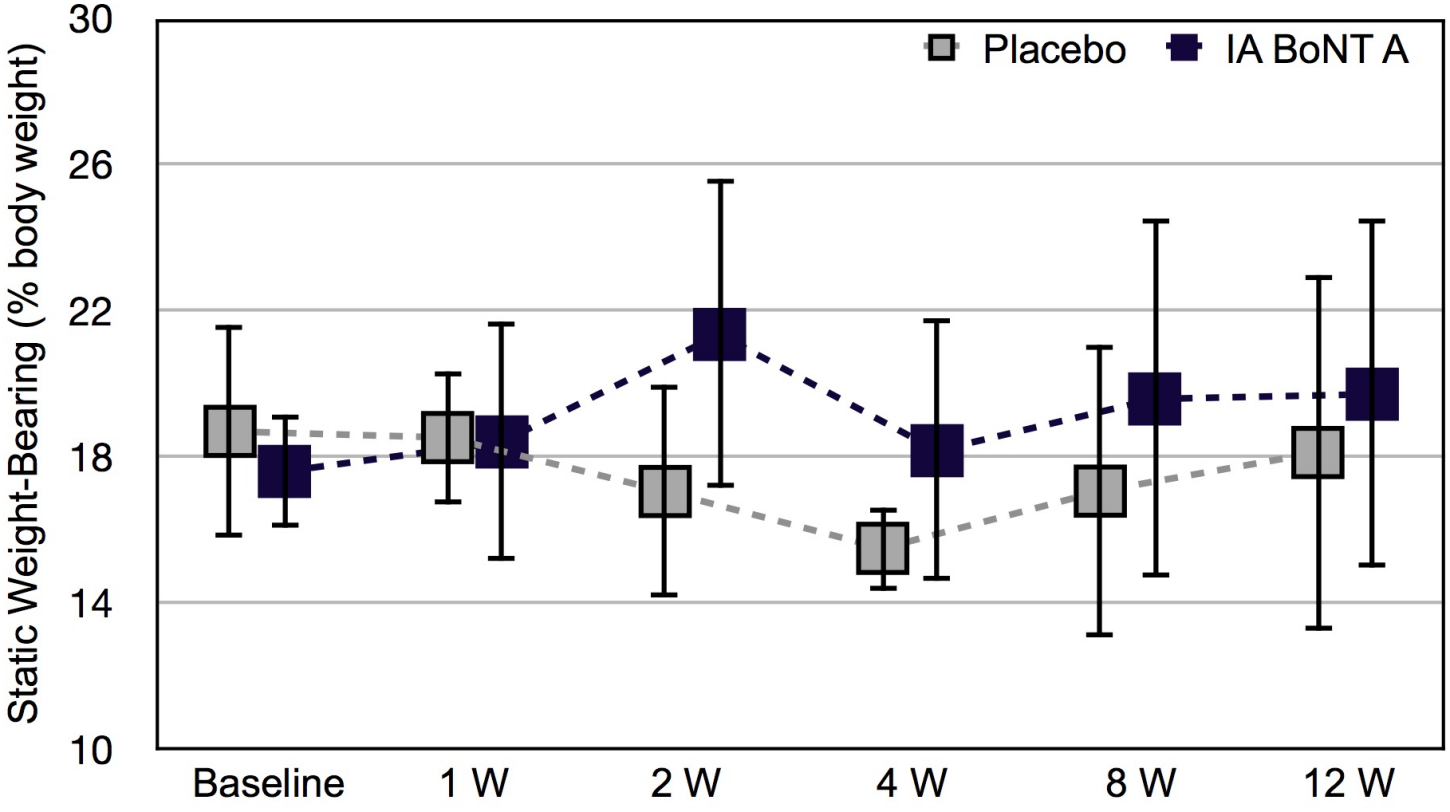
Static weight-bearing after intra-articular botulinum toxin A or placebo. Static weight-bearing (presented as mean and SD) of hind limbs of six healthy Beagle dogs. Hind limbs of each dog were randomized to receive either intra-articular botulinum toxin A or placebo (0.9% saline). Baseline, before the injections; W, week.

The static weight-bearing decreased from baseline in the placebo-injected limbs and increased from baseline in the IA BoNT/A-injected limbs during the study (Fig 2) (S2 Table), but the change from baseline was not statistically significant within the limb (mean estimated change in weight-bearing presented as percentage of body weight 1.31% units, 95% CI -1.83–4.45% units, P = 0.406 for IA BoNT A injected limbs; -0.92% units, -4.07–2.22 kg, P = 0.556 for IA placebo injected limbs). The difference between the groups in the change from baseline during the study was significant (*P* = 0.013).

#### Painless range of motion of joint

**Fig 3:**
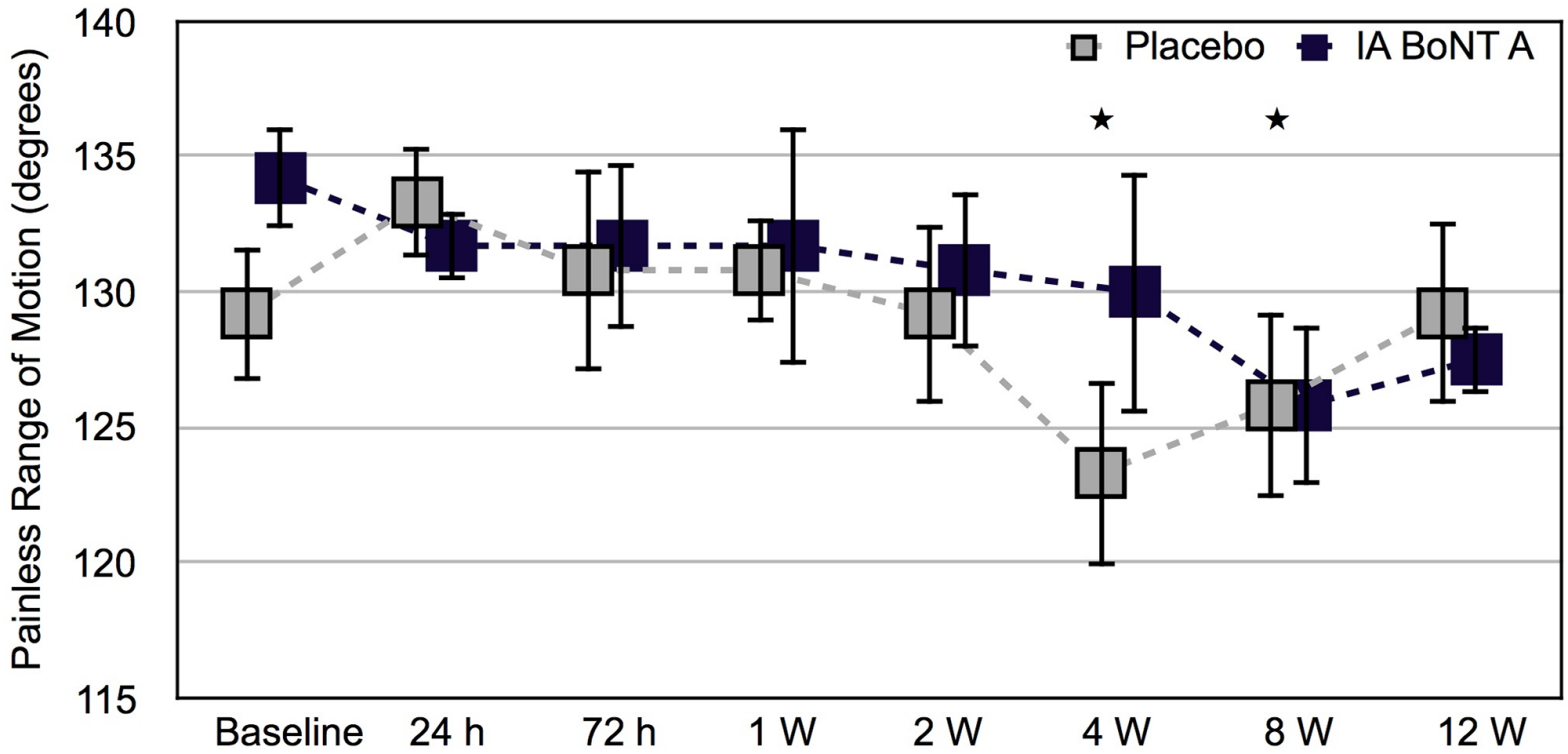
Painless range of motion of stifle joints after intra-articular botulinum toxin A or placebo. Painless range of motion (presented as mean and SD) of stifle joints of six healthy Beagle dogs. Hind limbs of each dog were randomized to receive either intra-articular botulinum toxin A or placebo (0.9% saline). ★ = Differs significantly from baseline, P = 0.001 for 4 W, P = 0.017 for 8 W. Baseline, before the injections; h, hour; W, week.

No significant difference emerged in the change from baseline in the painless range of motion between the BoNT/A- and placebo-injected stifle joints during the study (*P* = 0.150) (Fig 3) (S3 Table). However, the painless range of motion decreased significantly from baseline in the placebo-injected joints (mean estimated decrease -3.5°, 95% CI -5.9°–-1.0°, *P* = 0.007).

#### Pain in palpation of joint

No pain was detected in any of the joints during the study.

### Evaluation of spread of toxin

#### Neurological examination

Abnormal neurological examination findings were present in three dogs at baseline and in an additional two dogs during the study (Table 3).

**Table 3:** Neurological examination findings in healthy Beagle dogs (n = 6) after intra-articular botulinum toxin A and placebo.

		Timepoint						
	Baseline	24 h	72 h	1 W	2 W	4W	8 W	12 W
Dog 1	NO	NO	NO	NO	NO	Patellar ^A^^,^^B^↓↓	Patellar ^A^↓	NO
Dog 2	Extensor carpi radialis ^C^↓	Extensor carpi radialis ^C^↓	NO	NO	Facial sensation↓	Facial sensation ↓	NO	NO
Dog 3	Menace response↓	NO	NO	NO	Menace response ↓ Facial sensation ↓	NO	Menace response ↓	Menace response ↓
Dog 4	NO	NO	NO	NO	NO	Patellar ^B^↓	NO	NO
Dog 5	Patellar ^B^ ↓ Extensor carpi radialis ^C^↓	NO	NO	NO	NO	NO	NO	NO
Dog 6	NO	NO	NO	NO	NO	NO	NO	NO

^A^ in the limb injected with intra-articular botulinum toxin A.

^B^ in the limb injected with intra-articular placebo.

^C^ in front limb.

Baseline, before the injections; extensor carpi radialis, extensor carpi radialis reflex; h, hour; NO, no abnormal findings; patellar, patellar reflex; W, week; ↓ = mildly decreased; ↓↓ = severely decreased.

#### Electrophysiological recordings

There was no statistically significant difference between the IA BoNT/A and placebo limbs in the change from baseline in the MNCV, CMAP amplitudes, or RNS between (Table 4). Although there was a statistically significant decrease from baseline in the MNCVs, the MNCVs remained in the reference range published for dogs [48]. There was no statistically significant decrease from baseline in the CMAPS in the IA BoNT/A and placebo limbs, instead, the CMAPs increased during the study.

**Table 4:** Electrophysiological recording results in hind limbs of healthy Beagle dogs (n = 6) after intra-articular botulinum toxin A and placebo.

Variable	IA Injection	Timepoint					P-value	
		Baseline	2 W	4 W	8 W	12 W	Within Group	Between Groups
MNCV	BoNT/A	101.7 (12.2)	98.2 (7.1)	76.2 (5.3)	88.6 (6.0)	88.7 (3.6)	<0.001	0.468
	Placebo	100.4 (14.6	85.5 (4.8)	81.5 (4.4)	87.3 (2.4)	85.8 (4.7)	<0.001	
CMAP Amp Prox	BoNT/A	25.2 (4.2)	28.5 (2.4)	32.9 (2.9)	25.1 (2.9)	33.4 (4.6)	<0.001	0.537
	Placebo	23.2 (4.6)	27.8 (3.5)	33.5 (2.3)	30.6 (1.8)	30.0 (3.3)	<0.001	
CMAP Amp Dist	BoNT/A	22.2 (3.0)	23.5 (2.9)	29.6 (1.8)	21.7 (3.6)	26.1 (7.7)	<0.001	0.062
	Placebo	21.4 (4.2)	27.6 (2.3)	28.8 (1.8)	29.8 (2.3)	30.5 (4.2)	<0.001	
RNS Amp Max Decrement	BoNT/A	0.7 (0.5)	0.8 (0.8)	2.8 (2.4)	1.0 (0.8)	0.2 (0.2)	0.832	0.660
	Placebo	1.8 (1.8)	1.3 (0.9)	0.8 (0.5)	3.2 (1.6)	1.5 (0.8)	0.366	
RNS Area Max Decrement	BoNT/A	1.8 (0.8)	3.2 (1.1)	3.5 (1.8)	2. (1.2)	1.0 (0.5)	0.774	0.738
	Placebo	0.8 (0.7)	0.8 (0.3)	1.3 (0.6)	3.8 (0.8)	2.3 (0.8)	0.188	

Results are presented as mean (SE). Amp, amplitude; BoNT/A, botulinum toxin A; CMAP, compound muscle action potential; Dist, distal stimulation; IA, intra-articular; MNCV, motor nerve conduction velocity; placebo, 0.9% saline; Prox, proximal stimulation; RNS, repetitive nerve stimulation.

When the dogs were individually evaluated, the results of the electrophysiological recordings were normal in three dogs and abnormal in three dogs. The abnormal findings were the following:

Dog 1 had increased insertional activity in the supraspinatus and infraspinatus muscles at baseline. At 12 weeks after the injection, this dog had a CMAP amplitude of 8.66 mV in distal stimulation in the IA BoNT/A limb, which is lower than the calculated canine reference value (10.94 mV for proximal stimulation and 10.02 mV for distal stimulation of the peroneal nerve) [48].

Dog 2 had low CMAP amplitudes in distal stimulation in the IA BoNT/A-injected limb at 8 and 12 weeks after the injection (9.15 mV and 8.65 mV, respectively). This dog also had fibrillation potentials on the deep and superficial flexors of one front limb 12 weeks after the injection.

Dog 4 had low CMAP amplitudes in both proximal and distal stimulation in both hind limbs at baseline (6.20 mV and 7.13 mV, 6.80 mV and 5.12 mV for proximal and distal stimulation in the IA BoNT/A- and placebo-injected limbs, respectively). All of the electrophysiological recordings were considered normal in this dog at other examination time-points during the study.

Motor nerve conduction velocities were above the reference values published for dogs [48,50] in all dogs at all examination time-points. Repetitive nerve stimulation at 3 Hz did not show any abnormal response (i.e. >10% decrement [51]) at any examination time-point during the study. The rectal temperature of the dogs varied between 36.7C° and 38.4C° during the recordings, thus not affecting our results.

### Evaluation of cytological and histopathological adverse effects

#### Synovial fluid analysis

No changes of color, viscosity, or turbidity were detected in the SF samples during the study, except in one sample, which had severe blood contamination. The total and differential cell counts were in the reference range [52] in all samples except the one with blood contamination, which had a neutrophil percentage of 76. The sample was not included in the statistical model. The total cell count was calculated from only 25 out of 60 samples due to insufficient volume of SF, and therefore, was not included in the statistical analysis.

No significant difference was present in the SF neutrophil percentage between the BoNT/A- and placebo-injected joints during the study (*P* = 0.960).

#### Histopathological examination

General pathology revealed mainly changes associated with euthanasia and sampling directly postmortem (moderate splenic congestion in all dogs and scattered hypereosinophilic myocytes in dogs 2, 3, and 5) or incidental mild findings (mild lymphocytic or lymphoplasmacytic nephritis in dogs 1, 2, and 3). In addition, dog 1 had mild lymphoplasmacytic myocarditis. Scattered spheroids (median 5.5 spheroids /cross-section, range 1–11) were noted in the lumbar spinal cord of the dogs.

No macroscopic pathology was detected in any of the examined joints. No significant difference emerged in the scores regarding cartilage structure, synovial structure, or synovial inflammatory cell infiltrates between the BoNT/A- and placebo-injected joints (Table 5). The histopathological findings were few and scattered, although even very mild changes were recorded. No significant difference was found between the IA BoNT/A- and IA placebo-injected limbs in the histopathological changes related to muscle or nerve pathology (Table 6). The number of inflammatory cells was similar in the nerves adjacent to the BoNT/A- and placebo-injected stifle joints (median 4 cells per 5HPF, IQR 3–6 in the IA BoNT/A limbs; 4, 4–5 in the IA placebo limbs, *P* = 1.000).

**Table 5:** Histopathological changes in stifle joints of healthy Beagle dogs 12 weeks after intra-articular botulinum toxin A or placebo.

Variable	IA BoNT/A n = 6	IA Placebo n = 6	P-value
Cartilage structure	3 (0–3)	4 (2–7)	0.534
Synovial structure	3 (3–4)	3 (2–4)	0.593
Synovial infiltrates	1 (1–2)	2 (1–2)	0.638

Changes are presented as median and IQR.

IA BoNT/A, intra-articular botulinum toxin A; placebo, 0.9% saline.

**Table 6:** Histopathological changes in muscles and nerves of healthy Beagle dogs 12 weeks after intra-articular botulinum toxin A or placebo.

Sample	Variable	IA injection	Pathological findings		P-value	Odds ratio (95% CI)
			Present (number of dogs)	Absent (number of dogs)		
Muscles^A^	Cell size variation	BoNT/A	4	2	0.477	0.27 (0.00–20.83)
		Placebo	5	1		
	Angular fibers	BoNT/A	2	4	0.427	0.18 (0.00–31.45)
		Placebo	1	5		
	Ring fibers	BoNT/A	2	4	0.477	3.65 (0.05–277.07)
		Placebo	1	5		
Nerves^B^	Büngner bands	BoNT/A	2	4	0.292	5.00 (0.15–167.59)
		Placebo	4	2		
	Wallerian degeneration	BoNT/A	4	2	1.000	1.00 (0.03–28.95)
		Placebo	4	2		

^A^Evaluated in popliteal, vastus lateralis, and semimembranosus muscles.

^B^evaluated in sciatic, tibial, and saphenous nerves.

BoNT/A, botulinum toxin A; IA, intra-articular; placebo, 0.9% saline.

## Discussion

We investigated the safety of an IA BoNT/A injection by quantitatively evaluating the clinical, cytological, and histopathological adverse effects in six healthy laboratory Beagle dogs. Potential local and systemic spread of the toxin after an IA injection was also assessed. Our results support the hypothesis that IA BoNT/A does not produce significant clinical, cytological, or histopathological adverse effects. While the toxin may be spread from the joint, its effect seems to be mild.

### Evaluation of clinical adverse effects

Dose-dependent local weakness is a commonly reported adverse effect after an IM or subdermal BoNT/A injection [31,53–55]. It is thought to be a consequence of local spread of the toxin into adjacent tissues [5]. In a patient with arthritis, local muscle weakness would be especially undesirable as it would counteract the muscle strengthening exercise, which is an important part of OA treatment [8]. In addition to local weakness, injection site pain significantly reduces the compliance of IM and subdermal BoNT therapy in human patients [56–59]. Previously, both no joint pain [28] and mild pain of two days’ duration [44] has been reported in osteoarthritic dogs after IA BoNT/A.

We hypothesized that local weakness would affect both the movement and stance of our dogs, and to best assess this we measured both dynamic weight-bearing while the dogs were running and static weight-bearing while the dogs were standing. Dynamic weight-bearing was assessed with a force platform, which provides an objective and sensitive measurement of limb loading during motion in dogs [60,61]. It is currently the reference standard for evaluating treatment outcome in dogs with various orthopedic and neurological conditions [62–64]. The determination of a symmetry index is a method to evaluate the balance in dynamic limb loading based on right to left comparisons rather than absolute values of the measured forces, which are subject to effects of absolute and relative dog velocity and acceleration [61,65]. The static weight-bearing was evaluated with bathroom scales, an objective and sensitive method validated for dogs [46] and for hemiplegic human patients [66].

Our results suggest that local weakness and injection site pain are not a concern after 30 IU of IA BoNT/A in dogs. Instead, we detected a weight shift towards the IA BoNT/A-injected limbs in static weight-bearing and a decrease in the painless range of motion in the placebo-injected joints during the study. Arthrocentesis and electrophysiological recordings can be painful procedures and are therefore performed in sedated animals. Although we did not detect any pain in palpation of the joints in our dogs, the decreased weight-bearing in the IA placebo-injected limbs and the decrease in the range of motion in the IA placebo-injected joints might indicate that repeated arthrocenteses and electrophysiological recordings may cause mild, prolonged pain or other unpleasant sensations in healthy animals. If this hypothesis is true, BoNT/A may have alleviated these in the limb injected with the toxin.

The injection site pain associated with IM and subdermal injections has been attributed to reconstituting the drug with preservative-free saline [67–69] and to the low pH of the drug [70]. The pH of SF is higher than that of muscles and blood [71,72], which may explain not detecting pain in the IA BoNT/A injected joints in our study. In addition, we used preserved saline for the reconstitution of the BoNT/A.

### Evaluation of spread of toxin

Electrophysiological recordings detect disturbances in neuromuscular transmission and are therefore used in the diagnostics of botulism in human and veterinary patients [73,74]. In human patients with botulism, the typical electrophysiological findings include normal MNCV, decreased CMAP amplitude after a single stimulation with a further decline after repetitive slow-rate stimulation, and an increment in CMAP amplitudes at high-rate stimulation [73,75,76]. The most consistent finding of these is the decreased CMAP, which is seen in approximately 85% of patients with botulism [73]. A decrease in the CMAP amplitudes in RNS at slow-rate stimulation is seen infrequently, and an increment in the CMAP amplitudes at high-rate stimulation is seen in approximately 62% of patients [73]. Additionally, spontaneous activity may be detected in EMG [73,75]. Single-fiber EMG typically, but not consistently, reveals jitter and blocking [40,77].

There are only a few case reports describing the findings of electrophysiological recordings in dogs with botulism [51,78,79]. The CMAP amplitudes are decreased, and RNS at a low frequency rate (3 Hz) leads to a further decline in the CMAP amplitudes. Motor nerve conduction velocity is usually normal, but decreased velocity has also been reported. Additionally, EMG might be normal or may show spontaneous electrical activity [51,74,78,79].

In our study, the decrease of the CMAP amplitudes below the reference range in the IA BoNT/A-injected limbs after the injection (dogs 1 and 2) may suggest local spread of the toxin in these two dogs. Low CMAP amplitudes have been reported as the most consistent finding in human patients with botulism [73]. However, it is not known whether this is also true in dogs because few reports describe the electrophysiological findings in this species. Additionally, dog 2 had abnormal activity in EMG in one front limb during the study, which might indicate systemic spread. A previous case report describes markedly reduced CMAP amplitudes (mean 1.3 mV, SD ± 0.5 mV at admission and 1.9 mV ± 1.0 mV 7–15 days later) in dogs with generalized botulism [78], while in our study the low CMAP amplitudes measured after the IA BoNT/A injection in dogs 1 and 2 were only moderately decreased. Also, in dog 4 the CMAP amplitudes were low before any injections, but this might indicate that the dog had a subclinical, transient neuromuscular disorder at the beginning of the study.

The electrophysiological changes did not coincide with the abnormalities detected in the neurological examinations in our study. Previously, markedly low CMAP amplitudes have been detected at the same time with severely decreased or absent spinal reflexes, variable degree of cranial nerve dysfunction, and decreased or absent postural reactions in dogs with botulism [51,74,78,79]. In our study, four dogs had abnormalities in neurological examination after IA BoNT/A. Most of these abnormalities were mild and may stem from the fear or shyness of the examined dog. The decreased menace response in dog 3 was present already at baseline. The decreased patellar reflexes in dog 1 might be a result of the spread of the toxin, because withdrawal and patellar reflexes are considered more reliable in evaluation of the integrity and function of the reflex arches than extensor carpi radialis and tibialis cranialis reflexes [80]. On the other hand, decreased patellar reflexes are also seen in dogs with stifle joint disease. In addition, in dog 4 the patellar reflex decreased only in the placebo-treated limb during the study. Therefore, we consider it more likely that the decreased patellar reflexes detected after the injections arise from the repeated manipulations performed on the joints, not from the spread of the toxin. This is supported by the fact that electrophysiological recordings are a more sensitive method to detect spread of BoNT/A than clinical examination [40,81] and that in the previous studies on IA BoNT/A, no abnormalities have been detected in neurosensory testing in human patients [22,23,26], dogs [28,44], or horses [82].

Based on the results of the electrophysiological recordings, we conclude that IA BoNT/A may spread from the joint, but its clinical impact seems to be low.

### Evaluation of cytological and histopathological adverse effects

Intra-articular BoNT/A did not have any acute or delayed effects on the SF differential cell count in our study, which is in accordance with DePuy et al. (2007) [82], who reported normal SF analysis in two horses two weeks after IA BoNT/A.

In addition, our results suggest that 30 IU of IA BoNT/A does not cause pathological changes in the cartilage and synovium and does not lead to synovial inflammation in healthy stifle joints of dogs 12 weeks later. Also, no major pathological findings were detected in the complete autopsy.

The autopsy findings in patients with foodborne botulism are non-specific, except for gross diffuse muscular atrophy [83]. Spinal spheroids, which are focal axonal swellings associated with axonal degeneration and blockage of axonal transport, are commonly found in low numbers in adult and aged animals. Absolute numeric cut-offs for when spheroids indicate disease are not given, however, a local increase in spheroids can occur with spinal cord compression and vitamin E deficiency [84]. The spheroids noted in the lumbar spinal cord of the dogs in this study were randomly scattered within the white and gray matter and were not restricted to specific white tracts as would be expected in axonopathies of chronic intoxications. Findings indicative of a neuroaxonal dystrophy, such as spheroids mainly in the gray matter affecting the proximal axonal segment, were also not seen. In view of the lack of clinical signs of spinal dysfunction, we consider this finding incidental in our dogs. The mild lymphoplasmacytic myocarditis in the heart of dog 1 may be a consequence of a previous infection because no clinical signs of infection were detected during the study.

To the best of our knowledge, this is the first study to investigate the effects of this strong neurotoxin on articular cartilage in a healthy joint *in vivo*. Our findings are in accordance with a pilot study by DePuy et al. (2007) [82], which investigated the anti-inflammatory effects of IA BoNT/A in horses with experimentally induced inflammatory arthritis. In that study, no histopathological changes were detected in the synovium of two healthy control joints 15 days after IA BoNT/A. Our results are also in agreement with the *in vitro* findings of Henzel et al. (2008) [85], who demonstrated that adding BoNT/A to a culture of articular chondrocytes does not increase cell death in the culture. Previously, IA BoNT/A has shown chondroprotective, anti-inflammatory, and anti-fibrotic effects on articular cartilage and articular connective tissues in animal models of OA and acute inflammatory arthritis [86–88].

Intramuscular BoNT/A causes dose-dependent, long-lasting denervation atrophy in the injected muscle [41,42,89,90] and degeneration in the nerve innervating the muscle [43]. In addition, neurogenic atrophy of contralateral muscles and muscles very distant from the injection site has been reported after repeated IM injections, which is related to the spread of the toxin [42,91]. However, this has not been observed after a single IM injection [41,89]. In our study, there was no bilateral asymmetry and no significant difference in mild histopathological changes in the nerves and muscles between the IA BoNT/A- and IA placebo-injected limbs. This supports the conclusion that the possible spread of the toxin detected in the electrophysiological recordings is of minor clinical significance.

### Limitations

Several limitations apply to this study. One limitation is that only one concentration of the toxin was studied. The concentration to which the toxin is reconstituted (and thus the volume injected) affects the probability of diffusion from the injection site into adjacent structures, but there is no consensus on the optimal concentration. Injecting BoNT/A in a lower concentration and in a larger volume has been shown to enlarge the area of effect compared to injections of higher concentration and smaller volume [92–96]. In addition, lower concentration has been associated with stronger clinical effect [94], but no difference in clinical efficacy between low and high concentrations has also been reported [93,97]. Higher concentration has been associated with effect of longer duration but also with more adverse events [97]. We reconstituted the toxin to a concentration of 100IU/ml. In the joint, the toxin is likely to be diluted into the SF and thus the effect of the concentration might not be as important as in subdermal or IM injections. Our results apply only to onabotulinum toxin A, not to the other commercial BoNT/A preparations which differ in molecular weight, neurotoxin complexes, biological potency, immunogenicity, and dosing units [98].

Additionally, single-fiber EMG might have provided further information about the spread of the toxin in this study [77], but although single-fiber EMG has been described in healthy dogs [99], it has not, to the best of the author’s knowledge, been studied in dogs with botulism.

Furthermore, as cytological and histopathological findings did not differ between the IA BoNT/A and placebo-injected joints, more sophisticated techniques, such as SF metabolomics, could be considered to more thoroughly investigate the effects of the toxin inside the joint.

## Conclusion

Our results indicate that BoNT/A does not produce significant clinical, cytological, or histopathological adverse effects when administered IA into the stifle joint of healthy dogs. Based on the results of the electrophysiological recordings, the toxin may spread from the joint, but its clinical impact seems to be low.

## Supporting information

S1 Table Dynamic weight-bearing of hind limbs in healthy Beagle dogs after intra-articular botulinum toxin A and placebo. (DOCX)

S2 Table Static weight-bearing of hind limbs of healthy Beagle dogs after intra-articular botulinum toxin A or placebo. (DOCX)

S3 Table Painless range of motion of stifle joints of healthy Beagle dogs after intra-articular botulinum toxin A or placebo. (DOCX)

S1 File Dataset of the study. (XLSX)
