# Supplementary material for: Assessing adverse effects of intra-articular botulinum toxin A in healthy Beagle dogs: A placebo-controlled, blinded, randomized trial
Source: PLoS One. 2018 Jan 10;13(1):e0191043. doi: 10.1371/journal.pone.0191043 (PMC5761897; doi:10.1371/journal.pone.0191043)
Supplement: S1 Table — (DOCX) [file pone.0191043.s001.docx]

**S1 Table. Dynamic Weight-Bearing of Hind Limbs in Healthy Beagle dogs after Intra-Articular Botulinum Toxin A and Placebo.**

| **Variable** | **Timepoint** | | | | | **P-value** |
| --- | --- | --- | --- | --- | --- | --- |
|  | **Baseline** | **2 W** | **4 W** | **8 W** | **12 W** |  |
| Symmetry index (%) | -0.4 (2.8) | 3.4 (1.5) | -0.4 (2.7) | -0.7 (3.9) | -4.3 (2.6) | 0.106 |

Results are presented as mean (SE). Symmetry indices were calculated from peak vertical forces of hind limbs of six healthy beagle dogs at trot. 0 = dog is moving in perfect symmetry; values > 0 = more weight-bearing on botulinum toxin A–injected limb; values < 0 = more weight-bearing in placebo-injected limbs; baseline, before the injections; W, week.
