# Supplementary material for: Assessing adverse effects of intra-articular botulinum toxin A in healthy Beagle dogs: A placebo-controlled, blinded, randomized trial
Source: PLoS One. 2018 Jan 10;13(1):e0191043. doi: 10.1371/journal.pone.0191043 (PMC5761897; doi:10.1371/journal.pone.0191043)
Supplement: S2 Table — (DOCX) [file pone.0191043.s002.docx]

| **Variable** | **IA Injection** | **Timepoint** | | | | | | **P-value** | | |
| --- | --- | --- | --- | --- | --- | --- | --- | --- | --- | --- |
|  |  | **Baseline** | **1 W** | **2 W** | **4 W** | **8 W** | **12 W** | | **Within Group** | **Between Groups** |
| Static Weight-Bearing (%body weight) | BoNT/A | 17.6 (0.6) | 18.4 (1.3) | 21.4 (1.7) | 18.17 (1.5) | 19.6 (2.0) | 19.7 (2.0) | | 0.366 | 0.010 |
|  | Placebo | 18.7 (1.2) | 18.5 (0.7) | 17.1 (1.2) | 15.5 (0.5) | 17.06 (3.98) | 18.1 (2.0) | | 0.565 |  |

**S2 Table. Static Weight-Bearing of Hind Limbs of Healthy Beagle Dogs after Intra-articular Botulinum Toxin A or Placebo.**

Results are presented as mean (SD). Static weight-bearing of hind limbs of six healthy beagle dogs was evaluated with bathroom scales. BoNT/A, botulinum toxin A; IA, intra-articular; placebo, 0.9% saline; W, week.
