# Supplementary material for: Assessing adverse effects of intra-articular botulinum toxin A in healthy Beagle dogs: A placebo-controlled, blinded, randomized trial
Source: PLoS One. 2018 Jan 10;13(1):e0191043. doi: 10.1371/journal.pone.0191043 (PMC5761897; doi:10.1371/journal.pone.0191043)
Supplement: S3 Table — (DOCX) [file pone.0191043.s003.docx]

­­­ **S3 Table. Painless Range of Motion of Stifle Joints of Healthy Beagle Dogs after Intra-Articular Botulinum Toxin A or Placebo.**

| **Variable** | **IA Injection** | **Timepoint** | | | | | | | | **P-value** | |
| --- | --- | --- | --- | --- | --- | --- | --- | --- | --- | --- | --- |
|  |  | **Baseline** | **24 h** | **72 h** | **1 W** | **2 W** | **4 W** | **8 W** | **12 W** | **Within**  **Group** | **Between Groups** |
| Painless range of motion (degrees) | BoNT/A | 134.2 (1.5) | 131.7 (1.1) | 131.7 (2.5) | 131.7 (3.6) | 130.8 (2.4) | 130.0 (3.7) | 125.8 (2.4) | 127.5 (2.5) | 0.395 | 0.150 |
|  | Placebo | 129.2 (2.1) | 133.3 (1.7) | 130.8 (3.1) | 130.8 (1.5) | 129.2 (2.7) | 123.3 (2.8) | 125.8 (2.4) | 129.2 (3.5) | 0.007 |  |

Painless range of motion was evaluated by goniometry in awake dogs. Results are presented as mean (SE). BoNT/A, botulinum toxin A; h, hour; IA, intra-articular; placebo, 0.9% saline; W, week.
